# Supplementary material for: Causal Relationship between Aging and Anorexia Nervosa: A White-Matter-Microstructure-Mediated Mendelian Randomization Analysis
Source: Biomedicines. 2024 Aug 16;12(8):1874. doi: 10.3390/biomedicines12081874 (PMC11351342; doi:10.3390/biomedicines12081874)
Supplement: Supplementary file 1 [file biomedicines-12-01874-s001.zip › Supplementary Figure/Figure S4/Singe SNP plot of MR analysis for TL to ubm-b-1472.pdf]

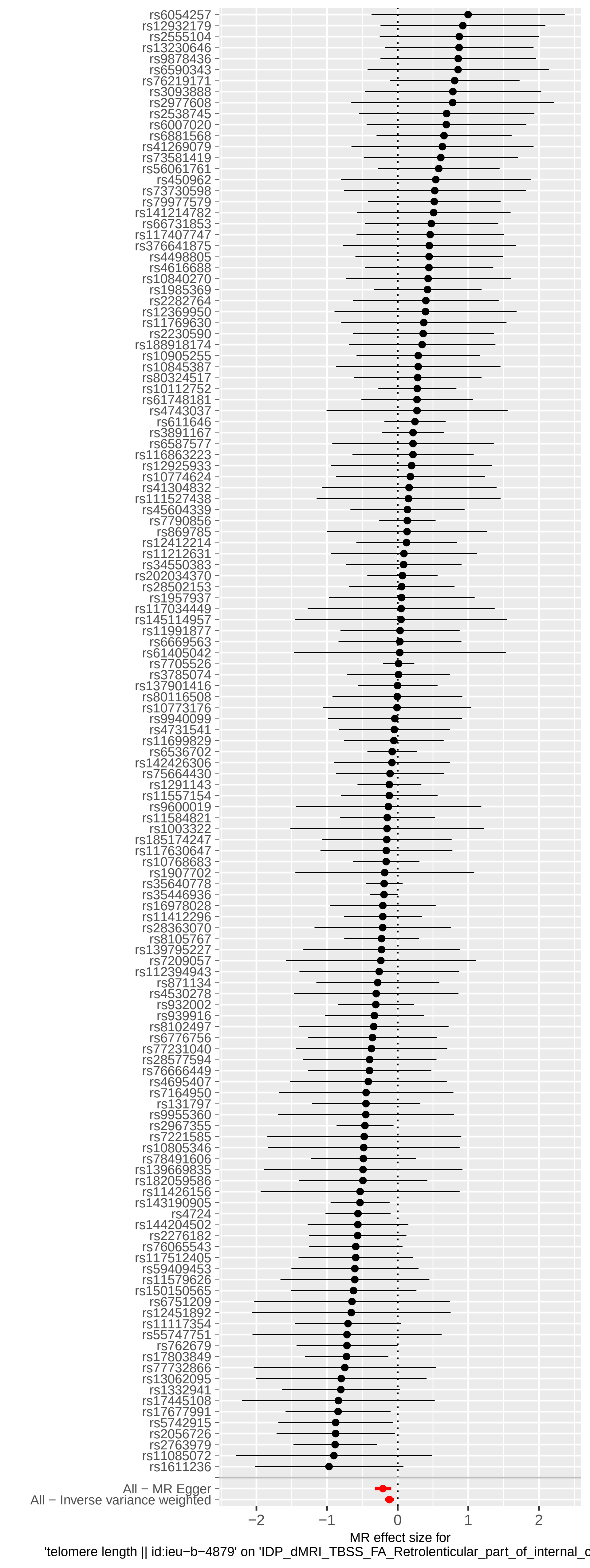
rs6054257
rs12932179
rs2555104
rs13230646
rs9878436
rs6590343
rs76219171
rs3093888
rs2977608
rs2538745
rs6007020
rs6881568
rs41269079
rs73581419
rs56061761
rs450962
rs73730598
rs79977579
rs141214782
rs66731853
rs117407747
rs376641875
rs4498805
rs4616688
rs10840270
rs1985369
rs2282764
rs12369950
rs11769630
rs2230590
rs188918174
rs10905255
rs10845387
rs80324517
rs10112752
rs61748181
rs4743037
rs611646
rs3891167
rs6587577
rs116863223
rs12925933
rs10774624
rs41304832
rs111527438
rs45604339
rs7790856
rs869785
rs12412214
rs11212631
rs34550383
rs202034370
rs28502153
rs1957937
rs117034449
rs145114957
rs11991877
rs6669563
rs61405042
rs7705526
rs3785074
rs137901416
rs80116508
rs10773176
rs9940099
rs4731541
rs11699829
rs6536702
rs142426306
rs75664430
rs1291143
rs11557154
rs9600019
rs11584821
rs1003322
rs185174247
rs117630647
rs10768683
rs1907702
rs35640778
rs35446936
rs16978028
rs11412296
rs28363070
rs8105767
rs139795227
rs7209057
rs112394943
rs871134
rs4530278
rs932002
rs939916
rs8102497
rs6776756
rs77231040
rs28577594
rs76666449
rs4695407
rs7164950
rs131797
rs9955360
rs2967355
rs7221585
rs10805346
rs78491606
rs139669835
rs182059586
rs11426156
rs143190905
rs4724
rs144204502
rs2276182
rs76065543
rs117512405
rs59409453
rs11579626
rs150150565
rs6751209
rs12451892
rs11117354
rs55747751
rs762679
rs17803849
rs77732866
rs13062095
rs1332941
rs17445108
rs17677991
rs5742915
rs2056726
rs2763979
rs11085072
rs1611236
All − MR Egger
All − Inverse variance weighted
−2
−1
0
1
2
MR effect size for
'telomere length || id:ieu−b−4879' on 'IDP_dMRI_TBSS_FA_Retrolenticular_part_of_internal_c
